# Attentional Biases toward Face-Related Stimuli among Face Dissatisfied Women: Orienting and Maintenance of Attention Revealed by Eye-Movement

**DOI:** 10.3389/fpsyg.2016.00919

**Published:** 2016-06-27

**Authors:** Hui Kou, Yanhua Su, Taiyong Bi, Xiao Gao, Hong Chen

**Affiliations:** ^1^Faculty of Psychology, Southwest UniversityChongqing, China; ^2^School of Management, Zunyi Medical UniversityZunyi, China

**Keywords:** dot probe paradigm, attention bias, facial attractiveness, facial dissatisfaction, eye-movement

## Abstract

The present study was aimed to examine attentional biases toward attractive and unattractive faces among face dissatisfied women. Twenty-seven women with high face dissatisfaction (HFD) and 27 women with low face dissatisfaction (LFD) completed a visual dot-probe task while their eye-movements were tracking. Under the condition of faces-neutral stimuli (vases) pairs, compared to LFD women, HFD women directed their first fixations more often toward faces, directed their first fixations toward unattractive faces more quickly, and had longer first fixation duration on such faces. All participants had longer overall gaze duration on attractive faces than on unattractive ones. Our behavioral data revealed that HFD women had difficulty in disengaging their attention from faces. However, there are no group differences in stimulus pairs containing an attractive and an unattractive face. In sum, when faces were paired with neutral stimuli (vases) HFD women showed an attention pattern characterized by orienting and maintenance, at least initially, toward unattractive faces but showed overall attention maintenance to attractive ones, but any attention bias wasn’t found in attractive - unattractive face pairs.

## Introduction

Facial attractiveness plays a central role in social activities, such as job hunting (Desrumaux et al., 2009), mate choice (McNulty et al., 2008), and interpersonal communication (Thornhill and Gangestad, 1999). Face dissatisfied individuals refer to those who show high levels of facial appearance concerns, produce negative cognition, and express negative emotion about their facial appearance, and therefore they will adopt relevant adjustments because of their unattractive faces (Rumsey and Harcourt, 2005). In this study, we used the face subscale of The Negative Physical Self Scale (Chen et al., 2006b) to identify women with high face dissatisfaction (HFD) and women with low face dissatisfaction (LFD)^1^.

Previous studies almost exclusively focused on body image dissatisfaction and eating disturbance, which revealed visual attention biases toward body-related stimulus based on sample differences in reaction time (RT), whether in Western culture (Ben-Tovim and Walker, 1991; Lee and Shafran, 2004) or in Eastern culture (Liang et al., 2008; Feng et al., 2010; Gao et al., 2011a,b, 2012). However, it was worth noting that Chinese adolescents and young adults expressed relatively more concerns about face appearance than about fatness (Chen et al., 2006b). What’s more, facial dissatisfaction could be regarded as an important part of body image dissatisfaction. Thus, it is of great importance to study spatial attention bias toward face-related stimulus with HFD based on the abovementioned research evidence.

Nevertheless, attention is a complicated cognitive process, including orientation, maintenance, disengagement and shift (Posner and Petersen, 1990). Moreover, each component of attention could dominate at a certain stage of attention Previous behavioral experiments using attention paradigms could only investigate one or two components of attention, such as attention orientation in the Stroop task (Ben-Tovim and Walker, 1991) and attention orienting and attention maintenance in the spatial cueing paradigm (Feng et al., 2010). The above paradigms could not reveal dynamical course of attention.

Researchers found that the eye-movement tracking technique provided a sensitive indicator for initial orienting, initial shift and maintenance of attention (Caseras et al., 2007). Some studies found that when viewing their own body, the eating disorders group focused less on their own self-defined “beautiful” body parts and more on their “ugly” parts than the control group did but showed the opposite pattern when viewing other people’s bodies (Jansen et al., 2005). Another study indicated that weight dissatisfied women directed their initial gaze toward fatness words, had a shorter mean latency of first fixation on both fatness and thinness words, had longer first fixation on fatness words but shorter first fixation on thinness words, and had shorter total gaze duration on thinness words (Gao et al., 2011b). Gao et al. (2012) further employed the technique of eye-movement in a dot probe experiment to explore attentional bias toward body-related pictures among females with weight dissatisfaction, and they found that an orienting-maintenance pattern of attention toward fatness-related pictures emerged among these women. It implied that individuals with body image dissatisfaction preferentially attended to body-related or fatness-related stimuli.

However, so far, attentional bias toward face-related stimulus among HFD females has not yet been studied, although facial dissatisfaction was also an important sub-component of body image dissatisfaction. Clinical research has revealed an attentional bias toward face-related stimuli among patients with body dysmorphic disorder (BDD) characterized by a distressing or impairing preoccupation with an imagined or slight defect in appearance that causes clinically significant distress or functional impairment (Didie et al., 2010). A study using the Stroop task indicated that, compared to controls, BDD patients were more distracted by face-related words (e.g., disfigured), with the greatest interference from positive face-related words (Buhlmann et al., 2002). However, another eye-movement study revealed that BDD patients showed increased selective attention to perceived defects in their own faces and unfamiliar faces (Grocholewski et al., 2012). These contradictory results might derive from inconformity in paradigms and stimulus. Using a dot probe task, researchers found that dysmorphic concern was positively correlated with attention to faces and attractive face-related images during the long stimulus presentation (1000 ms), whereas during the short stimulus presentation (200 ms), dysmorphic concern was positively correlated with disgusting images (Onden-Lim et al., 2012). We could conclude from this study that different attention components might play different roles at specific stages of attention course. More interestingly, considering that body image dissatisfaction has been considered to be a core feature of BDD (Cororve and Gleaves, 2001), studies of attentional bias toward faces among BDD patients could lay a solid foundation for attentional bias studies among HFD samples.

It is of great importance to study attentional biases toward faces among HFD individuals for two reasons. First, we may be able to further apply the model of body image dissatisfaction to faces. Secondly, we may be able to accordingly develop corresponding interventions to reduce distress for these people. Given that young females are more concerned with facial attractiveness than males in China (Chen et al., 2006a), we only recruited young females as our experimental participants. In the present study, we aimed to investigate the difference of spatial attentional biases between HFD women and LFD women using the eye movement-tracking technique. According to previous studies (Gao et al., 2011b, 2012), we hypothesized that in comparison with the LFD group, HFD women would be more likely to locate their initial fixation on unattractive faces, direct their first gaze faster to unattractive faces, have longer first fixation on unattractive faces, but have shorter first fixation on attractive faces, and have longer total gaze duration on unattractive ones. Regarding behavioral data, we predicted that HFD women might have difficulty in attentional disengagement from unattractive faces.

## Materials and Methods

### Participants

Our research sample was composed of 27 HFD women (age, *M* = 20.19, *SD* = 1.86) and 27 LFD female undergraduate students (age, *M* = 20.52, *SD* = 1.72) with ages ranging from 18 to 24 years old. The Negative Physical Self Scale-Face subscale (NPSS-Face) was employed to discriminate whether a participant had HFD or LFD (Gao et al., 2012). Only those who scored higher than 27.5 (HFD, *M* = 29.33, *SD* = 2.91) were considered to be HFD women; those who scored lower than 16.5 (LFD, *M =* 6.93, *SD* = 2.13) were regarded as LFD women. According to self-report, all of the participants were right-handed and had normal or corrected-to-normal vision and normal color vision, and none of them had a history of neurological or psychiatric illness.

### Materials

#### Facial Dissatisfaction Measure

The Negative Physical Self Scale (NPSS) has 48 items and is comprised of five subscales, including general appearance, face, fatness, shortness and thinness concern (Chen et al., 2006b; Chen and Jackson, 2007). The 11-item facial concern subscale (NPSS-Face) assessing thoughts, feelings, and behaviors related to face concerns was used to identify HFD and LFD women. The items were rated from 0 (“not at all like me”) to 4 (“very much like me”). The NPSS-Face was reliable (*α* = 0.85), stable over 9 months among girls (*r* = 0.62) from middle school to high school and had satisfactory convergent and predictive validity among samples of adolescents and young adults (Chen et al., 2006b; Chen and Jackson, 2007). In this research, its Cronbach coefficient was *α* = 0.964.

#### Stimuli

**FIGURE 1:**
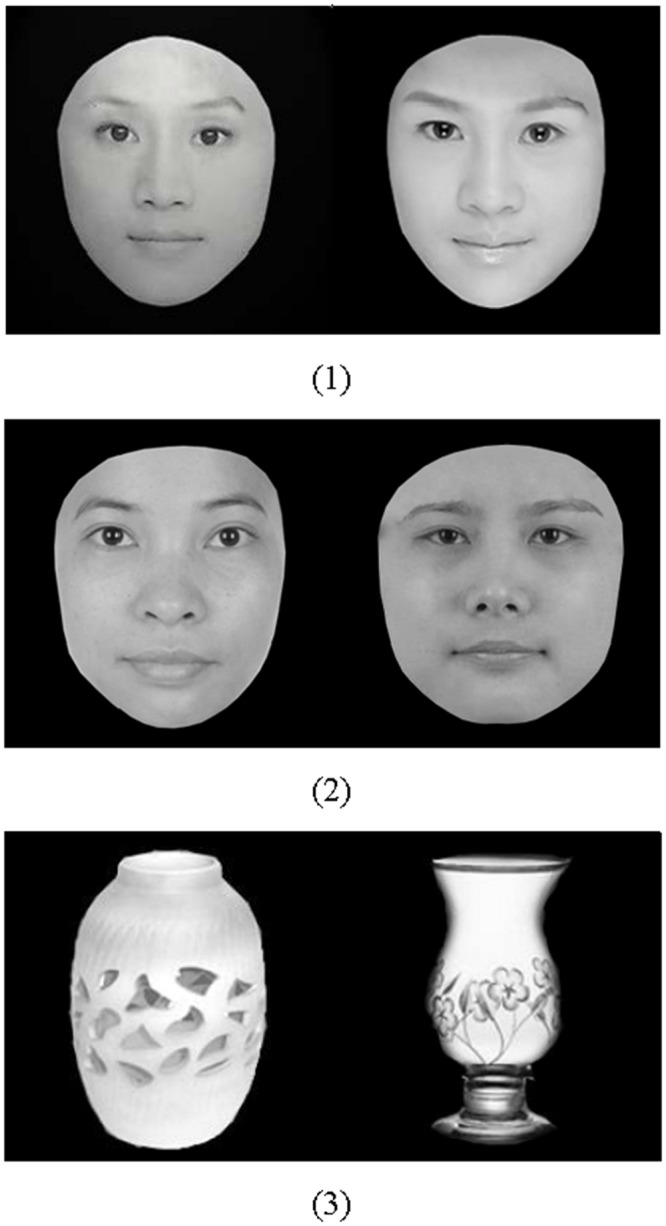
**Attractive faces (1), unattractive faces (2), and vase sample (3)**.

The photographic stimuli consisted of 18 attractive female face pictures, 18 unattractive female face pictures, and 36 neutral vase pictures. These pictures were adapted from a previous study (Zhang et al., 2010). They were cropped and sized into a uniform size (228^∗^228 pixel), which were displayed in black and white. Grayscale images were used. Furthermore, their hair, ear and neck were excluded by Photoshop. These standardized face pictures were assessed in arousal, pleasure and attractiveness by 45 undergraduate females who did not participate in the eye movement (EM) experiment and were required to respond on a five-point Likert scale from 1-*not a bit* to 5-*very*. Consequently, 18 attractive face pictures and 18 unattractive face pictures were selected. A paired-sample *t*-test on the two types of pictures revealed that there was no significant difference in arousal, *t* = -1.201, *df* = 17, *p* = 0.246 (unattractive faces *M* = 3.68, attractive faces *M* = 3.77; *MD* = -0.09, *SD* = 0.33), but there was a significant difference in pleasure, *t* = -4.20, *df* = 17, *p* = 0.001 (unattractive faces *M* = 2.78, attractive faces *M* = 3.22; *MD* = -0.44, *SD* = 0.10), and attractiveness, *t* = -6.50, *df* = 17, *p* < 0.001 (unattractive faces *M* = 2.72; attractive faces *M* = 3.33; *MD* = -0.62, *SD* = 0.40). Finally, 18 attractive-neutral picture pairs (A-N), 18 unattractive-neutral picture pairs (U-N), 18 attractive-unattractive picture pairs (A-U) and 18 neutral-neural picture pairs (N-N) were yielded. Examples of the stimuli are listed in **Figure 1**.

#### Apparatus

Eye movements were registered during the viewing period by an Eye Link II tracker (SR Research, Mississauga, ON, Canada) connected to a host computer. The sampling rate of the pupil-tracking mode was 1000 Hz, and the spatial resolution was 0.1°. The participants were seated approximately 60 cm in front of a 19-inch, 85-Hz monitor connected to a Pentium IV 3.2-GHz host computer and were provided with a chin rest to keep the viewing distance constant and minimize head movements. The participants were required to complete a standardized calibration procedure for EMs prior to the experiment, in which they were required to fixate on nine white dots randomly appearing on the black screen background in a 3 × 3 array.

### Procedure

The study was approved by the ethics committee for human research at Southwest University. Upon arrival at our laboratory, participants signed an informed consent form to learn about the aim of this study, which was to examine how EMs would be affected when viewing pictures. The task would last for about 30 min and participants were paid 10 Yuan as compensation for their time after the task. Subsequently, the participants were seated in front of the host computer and were asked to lay their chin on a chin-rest. They could regulate the height of the chin-rest accordingly to achieve a comfortable state.

Upon completion of standard calibration for their eye gaze, participants were required to complete the dot-probe task. At the beginning of each trial, a fixation cross was presented in the center of the screen for 1000 ms to prepare participants for the following procedures. Then, random picture pairs were immediately displayed for 1500 ms, followed by replacement of one of the pictures by a black dot (∙). Each picture pair was presented twice so that each picture location was counterbalanced by presenting them on both sides of the computer screen twice. When the fixation cross was presented, participants was asked to have to fixate the cross, viewed subsequent pictures and pressed one key (A) when the dots were located on the left side of the screen and another key (L) when the dots were on the right side as quickly as possible. Each probe appeared until a response was made within 5 s, followed by a blank screen for 300 ms. In total, 144 trials were performed in this experiment.

### Analysis

#### Eye Movement Data

Saccades that remained stable within a 1° visual angle for 100 ms or longer were defined as fixations to that position, the duration of which was recorded (Gao et al., 2011b). Fixations on either picture in each pair were effective when the following two conditions were satisfied: (i) Participants fixated in the central region (fixation cross) before picture onset, (ii) Saccades occurred at least 100 ms after picture onset and before picture offset (Fischer and Weber, 1993; Gao et al., 2011b). In this study, initial fixations were made on one of the pictures in 92.97% of the trials. The EM data were analyzed using the Eyenal Data Analysis Program (Applied Science Group 2000). There are four EM indices, including direction bias, first fixation latency bias, first fixation duration bias, and overall gaze duration bias. They can be calculated using the methods displayed in **Table 1** (Garner et al., 2006; Castellanos et al., 2009). Then, we used 2 (Group: HFD vs. LFD women) × 2 (Picture Type: attractive vs. unattractive faces) repeated measures ANOVA analysis with the abovementioned four EM indices as dependent variables.

**Table 1:** Calculation of eye movement (EM) indices.

EM indices	Calculation
Direction bias score	The number of trials in which first eye-movements were directed toward attractive or unattractive faces/total trials
First fixation latency bias score	The first fixation latency of attractive or unattractive faces – the first fixation latency of neutral pictures
First fixation duration bias score	The first fixation duration on attractive or unattractive faces – the first fixation duration on neutral pictures
Overall gaze duration bias score	The total time spent gazing at attractive or unattractive faces/the total time of gazing at face and neutral pictures

#### RT Data

According to a previous study (Koster et al., 2004), the attention disengagement index = [(BlDr+BrDl)/2-(NDl-NDr)/2]/2 (B: face pictures in the A-N and U-N pairs condition; D: the location of the probe; N: neutral pictures in the N-N condition; l: left; r: right). Positive values were indicative of orienting (i.e., faster RT to probes following face pictures than probes following neutral pictures), zero scores denoted no attentional bias, and negative scores reflected avoidance (i.e., slower RT to probes following face pictures than to those following neutral pictures) (Gao et al., 2011b). A 2(Group) × 2(Picture Type) repeated measures ANOVA analysis was also conducted with the scores of attention disengagement index as dependent variables.

## Results

### Eye-Movement Data

#### Direction Bias

Under the condition of A-N and U-N pairs, a 2 × 2 repeated measures ANOVA analysis was conducted with Group as the between-subjects factor (HFD vs. LFD) and Picture Type as the within-subjects factor (attractive vs. unattractive faces). The results demonstrated a significant main effect for Group, *F*(1,52) = 7.90, *p* = 0.007, η = 0.13, and significant interaction, *F*(1,52) = 4.58, *p* = 0.037, η = 0.08 (seen in **Table 2**). A simple effect analysis with Bonferroni adjustment suggested that HFD women directed their first fixations toward unattractive faces more often than attractive faces, *F*(1,52) = 4.52, *p* = 0.038, η = 0.08, and also toward unattractive faces more often than LFD women did, *F*(1,52) = 10.90, *p* = 0.002, η = 0.17. Additionally, in the condition of A-U pairs, independent sample *t*-test did not show any significant difference between HFD and LFD groups, *t* = -0.15, *df* = 50, *p* = 0.880.

**Table 2:** Eye-movement data of high face dissatisfaction (HFD) and low face dissatisfaction (LFD) group under the condition of attractiveness-neutral and unattractiveness-neutral pairs.

EM indices	Attractiveness-neutral pairs				Unattractiveness-neutral pairs				*F*(η)		
	HFD (*n* = 27)		LFD (*n* = 27)		HFD (*n* = 27)		LFD (*n* = 27)				
	Attractiveness	Neutral	Attractiveness	Neutral	Unattractiveness	Neutral	Unattractiveness	Neutral	Group	Picture type	Picture type × Group
					
	Mean (*SD*)	Mean (*SD*)	Mean (*SD*)	Mean (*SD*)	Mean (*SD*)	Mean (*SD*)	Mean (*SD*)	Mean (*SD*)			
A (%)	64.36 (12.75)		58.00 (10.57)		67.52 (13.15)		56.67 (10.91)		7.90^∗∗^ (0.13)	0.75 (0.01)	4.58^∗^ 0.08)
B (ms)	335.90 (42.49)	329.44 (42.84)	319.39 (27.89)	328.25 (34.97)	335.10 (38.20)	347.81 (46.32)	325.32 (30.63)	319.27 (31.07)	0.16 (0.00)	0.22 (0.00)	8.53^∗∗^ (0.14)
C (ms)	352.74 (86.53)	301.43 (119.73)	389.57 (117.26)	338.62 (120.35)	364.62 (93.36)	285.98 (103.23)	379.35 (92.08)	361.72 (104.17)	1.19 (0.02)	0.02 (0.00)	8.39^∗∗^ (0.14)
D (%)	60.20 (8.22)		61.43 (7.28)		56.13 (9.12)		56.95 (8.02)		0.25 (0.01)	25.19^∗∗∗^ (0.33)	0.06 (0.00)

*A, direction bias; B, first fixation latency; C, first fixation duration; D, overall gaze duration bias. ^∗^*p* < 0.05, ^∗∗^*p* < 0.01, ^∗∗∗^*p* < 0.001.*

#### First Fixation Latency Bias

Under the condition of A-N and U-N pairs, we then investigated the first fixation latency bias using a similar 2 × 2 repeated measures ANOVA. The results only revealed a significant interaction effect, *F*(1,52) = 8.53, *p* = 0.005, η = 0.14 (seen in **Table 2**). A further simple effect with Bonferroni adjustment examination indicated that HFD women obviously directed their first fixation toward unattractive faces more quickly than LFD women did, *F*(1,52) = 5.68, *p* = 0.021, η = 0.10, and also directed unattractive faces more quickly than they did the attractive faces, *F*(1,52) = 5.74, *p* = 0.020, η = 0.10. In addition, an independent sample *t*-test in A-U pairs revealed that there was no significant difference between groups, *t* = 1.32, *df* = 50, *p* = 0.19.

#### First Fixation Duration Bias

Under the condition of A-N and U-N pairs, a 2 × 2 repeated measures ANOVA revealed that the interaction effect was pronounced, *F*(1,52) = 8.39, *p* = 0.006, η = 0.13 (seen in **Table 2**). Simple effect analysis with Bonferroni adjustment indicated that HFD women were prone to fixate their initial gaze longer on unattractive faces than attractive ones, *F*(1,52) = 3.79, *p* = 0.057, η = 0.07, whereas LFD women showed the opposite mode, *F*(1,52) = 4.62, *p* = 0.036, η = 0.08. Moreover, a difference between groups emerged only with unattractive faces, *F*(1,52) = 5.92, *p* = 0.018, η = 0.10. In all, HFD women showed initial attention maintenance to unattractive faces, whereas LDF women merely focused on attractive faces. At last, under the condition of A-U pairs, an independent sample *t*-test revealed that there was no significant difference between HFD and LFD groups, *t* = 1.43, *df* = 50, *p* = 0.158.

#### Overall Gaze Duration Bias

Under the condition of A-N and U-N pairs, total gaze duration scores were analyzed using a 2 × 2 repeated measures ANOVA to investigate attention maintenance. It was found that only the main effect of Picture Type was significant, *F*(1,52) = 25.19, *p* < 0.001, η = 0.33 (seen in **Table 2**), suggesting that all participants fixated significantly longer on attractive faces than unattractive faces. Ultimately, an independent sample *t*-test did not show any significant difference between groups in A-U pairs, *t* = 0.25, *df* = 50, *p* = 0.803.

### RT Data

**FIGURE 2:**
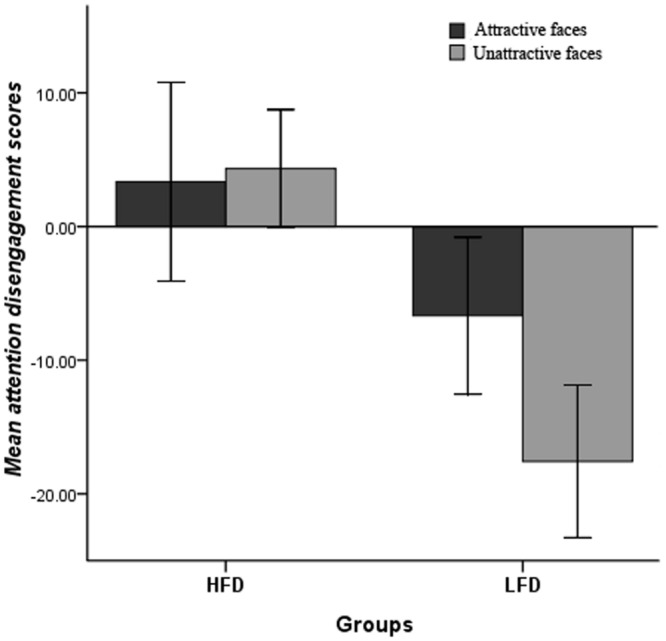
**The mean attention disengagement scores of under the condition of attractiveness-neutral and unattractiveness- neutral pairs among HFD and LFD women (error bar represents 95% confidence intervals level)**.

The attention disengagement scores were analyzed using a 2 × 2 repeated measures ANOVA under the condition of A-N and U-N pairs. The main effect of Group and the interaction effect reached significance, *F*(1,52) = 25.65, *p* < 0.001, η = 0.33, *F*(1,52) = 5.17, *p* = 0.027, η = 0.09 (seen in **Figure 2**). A simple effect analysis with Bonferroni adjustment indicated that the attention disengagement scores of HFD women, whether on attractive faces or on unattractive ones, were greater than the attention disengagement scores of LFD women, *F*(1,52) = 4.72, *p* = 0.034, η = 0.08, *F*(1,52) = 38.98, *p* < 0.001, η = 0.43; however, only the attention disengagement scores of attractive faces among LFD women were greater than the attention disengagement scores of unattractive faces, *F*(1,52) = 8.68, *p* = 0.005, η = 0.14, whereas there was no significant difference among HFD women, *F*(1,52) = 0.07, *p* = 0.790, η = 0.001. Compared to LFD women, HFD women showed attention maintenance to both unattractive and attractive faces.

In addition, the attentional disengagement index of unat tractive faces in A-U pairs was analyzed using an independent sample *t*-test, demonstrating no significant difference in groups, *t* = 0.74, *df* = 52, *p* = 0.315 (**Table 3**).

**Table 3:** Reaction time (in ms) for probe among high face dissatisfaction (HFD) and low face dissatisfaction (LFD) women.

		HFD		LFD	
Face pictures position	Probe position	*M*	*SD*	*M*	*SD*
**Attractiveness-neutral**					
Left	Left	511	105	512	82
Left	Right	520	122	495	85
Right	Left	524	125	521	105
Right	Right	484	109	494	80
**Unattractiveness-neutral**					
Left	Left	520	119	529	99
Left	Right	501	497	497	70
Right	Left	520	132	506	103
Right	Right	494	108	487	74

## Discussion

The purpose of the study was to assess spatial attention biases among HFD and LFD women by directly tracking their EM. To the best of our knowledge, this is the first experimental study to examine attention orienting-avoidance and disengagement-maintenance to attractive/unattractive faces among HFD and LFD women. Our hypothesis was partly supported by our results. Compared to LFD women, HFD women were more likely to locate their initial fixation on unattractive faces, directed their first gaze faster to them, and had longer first fixation on them, consistent with our hypothesis, but HFD women did not have shorter first fixation on attractive faces or longer total gaze duration on unattractive faces.

### Attention Orienting: Direction Bias and First Fixation Latency Bias

Regarding the A-N and U-N pairs, HFD women directed their first fixations toward both types of faces more often than the LFD group did and had more initial fixations on unattractive faces than attractive faces, which proved our hypothesis. Furthermore, HFD women directed their first fixation toward unattractive faces faster than LFD women did and directed their first fixation towards unattractive faces more quickly than they did the attractive faces, which also supported our hypothesis. These results suggested attention orienting of unattractive faces among HFD women. Consistent with this study, some previous researchers found that weight dissatisfied female showed similar biases in initial orientation characterized by more frequent and faster first fixations on fatness-related information (e.g., fat words and fat pictures) than on neutral stimuli (Gao et al., 2011b, 2012). Specifically, in an ERP study, Gao et al. (2011a) claimed that the early anterior N100 and bilateral parietal and occipital N170 amplitudes elicited by fatness-related words were larger than those elicited by thinness-related and neutral words among weight dissatisfied females. In addition, the amplitudes of N170 and N100 were larger for the negative words because the females were influenced by the valence of emotion (Doallo et al., 2007). According to the cognitive-behavioral model concerning body image dissatisfaction proposed by Vitousek and Hollon’s (1990), attention bias toward body-related stimuli arose from underlying maladaptive self-schemata associated with shape and weight. Furthermore, Perpiñá et al. (1993) found that individuals with maladaptive body schemata were different from others in several ways, including facilitation of attention and memory processing for schema-consistent or schema-related stimulus (e.g., fatness-related or body-related stimulus) (Perpiñá et al., 1993). Based on this model, we guessed that spatial attentional bias toward unattractive faces might derive from underlying maladaptive self-schemata associated with facial attractiveness, which could facilitate the attentional processing of unattractive faces among HFD women.

Concerning A-U pairs, all participants did not show any direction bias and first fixation latency bias toward unattractive faces. One reason might be that faces are generally attention-grabbing (Ro et al., 2001; Mack et al., 2002; Theeuwes and Van der Stigchel, 2006; Bindemann et al., 2007). When faces are presented along with vases, the face might initially capture attention, while the attractiveness or unattractiveness of the face can further modulate the overt attentional selection of the face. When two faces are presented, this might be a more complex process, because the available attentional resources are more strongly distracted by the other, concurrent face. Consequently, when an attractive face and an unattractive face are displayed simultaneously on a computer screen, participants did not show attention orienting or avoidance to any faces in the condition of A-U pairs.

### Attention Maintenance: First Fixation Duration Bias and Overall Gaze Duration Bias

Under the condition of A-N and U-N pairs, HFD women showed initial attention maintenance to unattractive faces, whereas LDF women merely focused on attractive faces. Our hypothesis was partly supported because attention avoidance to attractive faces among HFD women did not emerge in our study. However, the result was in line with a previous study (Gao et al., 2012), reporting that attention avoidance of thin body pictures was not found among weight dissatisfied females. In addition, our result here was consistent with a previous study investigating BDD individuals. They implied that during a long stimulus presentation (1000 ms), individuals with dysmorphic concern attended to both faces and face-related images, whereas during a short stimulus presentation (200 ms), they focused on disgusting images (Onden-Lim et al., 2012). Furthermore, similar to a previous study with weight dissatisfied female (Gao et al., 2011b), HFD women were indeed prone to focus longer on unattractive faces than attractive faces, which may indicate that once attention was captured by unattractive faces in the early stages of attentional processing, HFD women might have difficulty, at least initially, in disengaging from unattractive faces. Fox et al. (2002) considered that attentional bias may consist of two phases. In the first orientation phase, individuals are sensitive to threatening stimulus and attention is drawn to the threat stimulus. Second, threatening stimulus could influence the maintenance of attention or the participants’ ability to carry out attention disengagement (Fox et al., 2002). Accordingly, HFD women may regard unattractive faces as threatening stimulus and have difficulty in disengaging attention from unattractive faces. Evidence from behavioral data, in which HFD women had difficulty in disengaging from unattractive faces in the condition of A-N and U-N pairs, also proved this observed phenomenon. However, in the condition of A-U pairs, both EM data and behavioral data showed there was no difference between HFD and LFD women.

Analyses for overall fixation duration bias indicated that both groups had overall attention maintenance to attractive and unattractive faces relative to neutral stimulus. Moreover, both groups had more difficulty disengaging their overall attention from attractive faces than unattractive faces. Although the result was incongruent with previous research (Gao et al., 2012) indicating that weight dissatisfied women had overall gaze duration bias toward fat body-related pictures, some researchers found that faces had an advantage in retaining attention over other stimulus categories (Bindemann et al., 2005). Human beings are born to prefer face stimulus (Johnson et al., 1991; Valenza et al., 1996; Slater and Quinn, 2001), because faces convey social and biological implications, such as identity (age, gender etc.) and sex. Therefore, all participants fixated their attention on faces, whether they were attractive or not. Moreover, researchers found that people showed preference for attractive faces as early as during infancy (Langlois et al., 1990; Rubenstein et al., 1999). Moreover, behavioral studies have revealed that both HFD and LFD women focused longer on attractive faces than unattractive faces (Aharon et al., 2001). Brain imaging studies have revealed that attractive faces possess rewarding value and activated reward circuit and emotion-related brain regions, such as the OFC, amygdala and basal ganglia (O’Doherty et al., 2003). This could explain why HFD women showed overall attention maintenance, instead of avoidance, to attractive faces.

### Limitation and Prospection

Despite some interesting findings, this research contains several limitations. Firstly, although the study focused on undergraduate females with facial dissatisfaction, the findings may not generalize to undergraduate male and adolescent samples. Therefore, in future research, replications among samples from other age and gender groups are needed to assess the consistency and difference of such an attention model across gender and age. Secondly, the findings may not apply completely to non-Chinese samples. Chen and Jackson (2006), using a lexical decision task with subliminal priming, found that American participants with weight dissatisfaction judged self-primed positive body words more slowly than self-primed negative body words, while participants with relatively positive body image showed a contrary tendency. However, the matched Chinese participants did not show such a trend (Chen and Jackson, 2006). It is possible that there exists cultural diversity in attentional bias toward face pictures among HFD groups. Hence, extensions to samples of other cultural backgrounds are also one of our future research directions. Lastly, face pictures were displayed in black/white but not in color. Given that colored pictures are ecologically more valid, future studies employing colored pictures may promote the ecological validity of our findings.

## Conclusion

In summary, HFD women first showed attention orienting toward unattractive faces but did not show attention avoidance to attractive pictures. Additionally, HFD women, similar to LFD women, displayed difficulty in overall attention disengagement from attractive faces, but there weren’t any attention bias found in attractive – unattractive face pairs.

## Author Contributions

HK and YS conceived of the study, participated in its design and coordination, performed the measurement and drafted the manuscript together; HC participated in the design and interpretation of the data; TB participated in revising the manuscript critically for important intellectual content; XG participated in the statistical analysis. All authors read and approved the final manuscript.

## Conflict of Interest Statement

The authors declare that the research was conducted in the absence of any commercial or financial relationships that could be construed as a potential conflict of interest.

## Footnotes

1 High face dissatisfaction refers to high face dissatisfaction, and LFD refers to low face dissatisfaction.
